# Phylogenomics Resolves the Evolution of Sternorrhyncha (Insecta: Hemiptera)

**DOI:** 10.1002/ece3.72636

**Published:** 2025-12-05

**Authors:** Shiyu Du, Yehao Wang, Yuang Li, Chenyang Cai

**Affiliations:** ^1^ College of Life Sciences Nanjing Normal University Nanjing China; ^2^ State Key Laboratory of Palaeobiology and Stratigraphy Nanjing Institute of Geology and Palaeontology, Chinese Academy of Sciences Nanjing China; ^3^ Nanjing College University of Chinese Academy of Sciences Nanjing China; ^4^ The Key Laboratory for Silviculture and Conservation of Ministry of Education Beijing Forestry University Beijing China

## Abstract

Sternorrhyncha, one of the four major suborders of Hemiptera, represents a phytophagous insect clade comprising nearly 18,000 described species. While recent phylogenomic studies have made significant efforts to elucidate the internal phylogenetic relationships within this group, substantial controversies persist regarding both the higher‐level phylogeny and particularly the identification of the earliest‐diverging lineage. To investigate the phylogenomics of Sternorrhyncha and to reveal the reasons behind phylogenetic conflicts, this study utilizes the available genomic and transcriptomic data to reconstruct the Sternorrhyncha phylogeny and evaluates the impact of alternative evolutionary models on deep‐node resolution. A well‐supported backbone topology, (Aleyrodoidea, (Psylloidea, (Coccoidea, Aphidoidea))), was consistently recovered across multiple analytical approaches in our study. This study provides critical evidence for resolving the long‐standing phylogenetic controversies within this taxon.

## Graphical abstract

Phylogeny of Sternorrhyncha based on Matrix2 under PMSF (LG+C60+F+R) model.

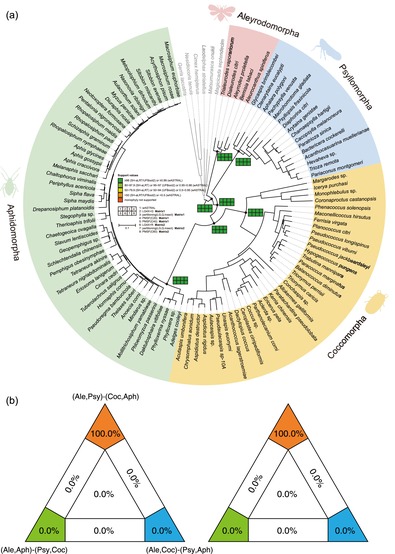

## 1 Introduction

Hemiptera boasts an extensive evolutionary history, with its remarkable morphological adaptations enabling the colonization of diverse habitats and exploitation of varied food sources. Today, it stands as one of the most specious and morphologically diverse insect orders, comprising over 100,000 described species (Li et al. 2017; Drohojowska et al. 2020). Extant Hemiptera are divided into four suborders: Sternorrhyncha, Auchenorrhyncha, Coleorrhyncha, and Heteroptera. The phylogenetic relationships among these suborders have been extensively studied, with Sternorrhyncha consistently recovered as the sister group to the remaining Hemiptera, which has been strongly supported by both morphological and molecular evidence (Bourgoin and Campbell 2002; Misof et al. 2014; Li et al. 2017).

Sternorrhyncha, a group of minute phytophagous insects with piercing‐sucking mouthparts, includes four extant superfamilies: Aphidoidea, Coccoidea, Aleyrodoidea, and Psylloidea, with approximately 18,000 described species distributed globally (Drohojowska et al. 2020; Liu et al. 2024). Traditional morphological studies support a sister‐group relationship between Coccoidea + Aphidoidea and Aleyrodoidea + Psylloidea (von Dohlen and Moran 1995; Drohojowska et al. 2020). Molecular phylogenetic analyses have provided new insights into the backbone phylogeny of Sternorrhyncha while introducing new challenges. The monophyly of the Coccoidea + Aphidoidea clade has been consistently supported across studies without controversy (Liu et al. 2024). In contrast, the phylogenetic positions of Psylloidea and Aleyrodoidea exhibit remarkable instability, varying with datasets and analytical methods (von Dohlen and Moran 1995; Misof et al. 2014; Zhou et al. 2022; Liu et al. 2024).

Two primary hypotheses have emerged: (1) (Psylloidea, (Aleyrodoidea, (Coccoidea, Aphidoidea))) (Campbell et al. 1994; von Dohlen and Moran 1995; Cryan and Urban 2012) and (2) (Aleyrodoidea, (Psylloidea, (Coccoidea, Aphidoidea))) (Misof et al. 2014; Johnson et al. 2018; Kieran et al. 2019; Wang et al. 2019; Song and Zhang 2022). The former has been supported by Sanger sequencing and mitochondrial genome analyses (Campbell et al. 1994; von Dohlen and Moran 1995; Cryan and Urban 2012; Xiong et al. 2017), whereas most large‐scale phylogenomic studies favor the latter, positioning Aleyrodoidea as the earliest‐diverging lineage (Misof et al. 2014; Johnson et al. 2018; Kieran et al. 2019; Wang et al. 2019; Song and Zhang 2022). Intriguingly, a recent study by Liu et al. (2024), incorporating ultraconserved element (UCE) data from 101 transcriptomes and genomes, largely rejected the Aleyrodoidea‐first hypothesis, instead supporting the traditional morphological view.

In this study, we integrated 110 genomes and transcriptomes (including six outgroups) using single‐copy orthologous genes, with a focus on evaluating the utility of site‐heterogeneous models in resolving Sternorrhyncha phylogeny. Our analyses yielded a robust backbone phylogeny, shedding light on the evolutionary relationships within this ecologically and economically important insect group.

## 2 Materials and Methods

This study integrates a comprehensive dataset of 110 genomic and transcriptomic sequences, comprising five aleyrodoids, 16 psylloids, 49 aphidoids, 34 coccoids, and six outgroup species. All data were sourced from publicly available NCBI databases (available at https://www.ncbi.nlm.nih.gov/) and Figshare repository (Liu et al. 2024). Please refer to Table S1 for more details.

Paired‐end reads derived from transcriptomic raw data were assembled using SPAdes v4.4.0 (Prjibelski et al. 2020) with the ‘‐‐rna’ strategy. The assembled genomes were evaluated and annotated using BUSCO v5.5.0 (Manni et al. 2021) with the Hemiptera database from OrthoDB version 10 (creation date: 2024‐01‐08; *n* = 2510; see Table S1), under the parameter ‘‐m geno’ (Kriventseva et al. 2019). To identify more “complete” sequences, the standard deviation (*σ*) threshold of the mean universal single‐copy ortholog (USCO) length was adjusted to 2*σ* (Du et al. 2024). For transcriptomes, redundancy reduction was performed initially using CD‐HIT v4.8.1 with default settings (Li et al. 2002) and followed by a BUSCO analysis. It should be noted that BUSCO predictions may miss divergent exons (Waterhouse et al. 2017). Consequently, this can lead to inaccurate gene annotations and the inference of erroneous isoforms. The processes of redundancy reduction and subsequent alignment trimming may eliminate these genomic regions. Despite not directly evaluating the impact of these potential spurious alignment signals on the phylogeny of Sternorrhyncha, our congruent results from both concatenation and coalescent‐based analyses suggest that their influence on the overall conclusions is negligible.

Nucleotide (NT) sequences were aligned according to their amino acid (AA) translations using MACSE v2.06 with ‘The Standard Code’ to produce the corresponding aligned AA sequences (Ranwez et al. 2018). Alignment refinement was disabled by setting the maximum number of refinement iterations to zero (−max_refine_iter 0). The resulting AA alignments were subsequently trimmed with BMGE v1.12 using a stringent setting (−m BLOSUM62 ‐h 0.4) to minimize sequence heterogeneity and reduce computational demands (Criscuolo and Gribaldo 2010). Finally, loci exhibiting species occupancy greater than 75% and 90% were concatenated using FASconCAT v1.04 (Kück and Longo 2014) to generate matrices 1 and 2 (Matrix1: 698,389 AA from 2134 loci; Matrix2: 145,039 AA from 406 loci), respectively, for downstream concatenation analyses (Table 1).

**TABLE 1:** Summary of USCO amino acid matrices used for phylogenetic analyses.

Matrix	Average missing taxa per locus (%)	No. of loci	No. of sites	Missing sites (%)	Average locus length
Matrix1	14.61	2134	698,389	18.57	327.27
Matrix2	8.28	406	145,039	12.15	357.24

For coalescent analyses, each AA alignment was trimmed with BMGE using a less stringent configuration (−m BLOSUM62 ‐h 0.5) to retain a greater number of informative sites for gene tree reconstruction. Gene trees were inferred in IQ‐TREE v2.2.2.7 (Minh et al. 2020) under the LG4X model (Le et al. 2012), with node support values estimated from 1000 ultrafast bootstrap replicates (Hoang et al. 2018). The resulting gene trees were then integrated to infer the species tree using the weighted algorithm implemented in ASTER v1.15, under a multi‐species coalescent (MSC) model to reduce the influence of incomplete lineage sorting (Zhang et al. 2025).

For concatenation analyses, we applied a partitioned model to both Matrices 1 and 2. The optimal partitioning scheme and corresponding substitution models were determined using ModelFinder in IQ‐TREE with the ‘‐m MFP + MERGE’ option (Chernomor et al. 2016; Kalyaanamoorthy et al. 2017). To further account for site‐specific compositional heterogeneity driven by biochemical constraints (Feuda et al. 2017), we additionally evaluated mixture models. Specifically, LG4X+R models were first tested for both matrices in IQ‐TREE (Le et al. 2012). Subsequently, C20‐PMSF and C40‐PMSF models were assessed for Matrix1, and C60‐PMSF for Matrix2, using the LG4X+R topology as a guide tree to reduce computational burden (Wang et al. 2018). Nodal support in the maximum‐likelihood (ML) analyses was evaluated using 1000 replicates of the SH‐like approximate likelihood ratio test (Guindon et al. 2010) and ultrafast bootstrap (Hoang et al. 2018). All trees were visualized and edited with FigTree v1.4.4 (https://github.com/rambaut/figtree.git) and iTOL v6 (Letunic and Bork 2024).

To infer the strength of phylogenetic signal and to assess the stability of our phylogeny, the four‐cluster likelihood mapping (FcLM) approach (Strimmer and von Haeseler 1997) was applied to all two matrices in IQ‐TREE. The FcLM analyses were conducted under the PMSF models (C40‐PMSF for Matrix1, and C60‐PMSF for Matrix2).

## 3 Results

All phylogenetic analyses in this study were conducted using AA sequences of USCO genes. This approach was adopted based on extensive evidence demonstrating the superiority of AA data for resolving deep phylogenetic relationships (Simion et al. 2017; Philippe et al. 2019; Williams et al. 2020). Compared to NT data, AA sequences effectively mitigate systematic errors arising from saturation effects, rate heterogeneity, and compositional heterogeneity (Wang et al. 2023). Besides, we implemented a multi‐model analytical framework, with explicit emphasis on site‐heterogeneous models and the MSC model, to account for potential site‐specific compositional heterogeneity and incomplete lineage sorting (ILS) within our dataset. Notably, all analyses recovered highly consistent topologies across distinct evolutionary models.

**FIGURE 1:**
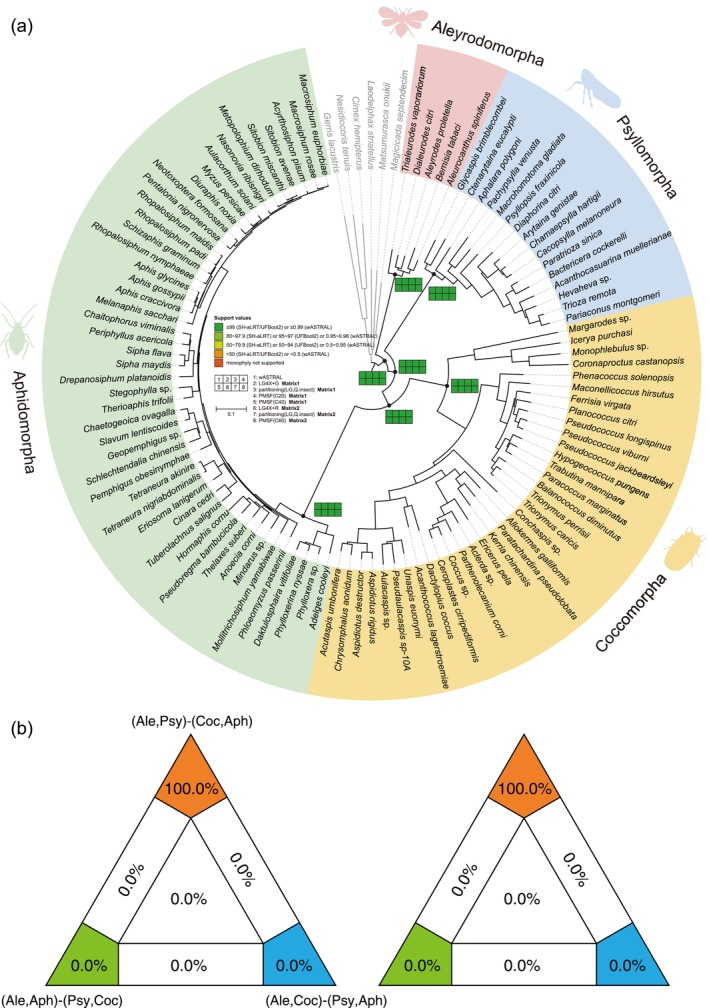
(a) Phylogeny of Sternorrhyncha based on Matrix2 under C60‐PMSF (LG+C60+F+R) model. Node supports from other analyses are also indicated by the colored squares (The node supports of each phylogenetic tree are shown in Appendix A). Image silhouettes sourced from PhyloPic (https://www.phylopic.org/), and contributed by Yuang Li. (b) Four‐cluster likelihood mapping (FcLM) analyses based on Matrix1 (left) and Matrix2 (right). Voronoi cells are areas, in which quartets show predominant or maximal support for either of the three unrooted topologies T1, (Aleyrodomorpha + Psyllomorpha) and (Coccomorpha + Aphidomorpha); T2, (Aleyrodomorpha + Aphidomorpha) and (Psyllomorpha + Coccomorpha); and T3, (Aleyrodomorpha + Coccomorpha) and (Psyllomorpha + Aphidomorpha).

In the higher‐level phylogeny of Sternorrhyncha, all four superfamilies, Aleyrodoidea, Psylloidea, Aphidoidea, and Coccoidea, were robustly recovered as monophyletic with maximal support (Figure 1a; UFBoot = 100, SH‐aLRT = 100, wASTRAL = 1). Their interrelationships were consistently resolved as: (Aleyrodoidea, (Psylloidea, (Aphidoidea, Coccoidea))).

Within Psylloidea, coalescent‐based analyses strongly supported the monophyly of Aphalaridae (wASTRAL = 1), placing it as the earliest diverging lineage, followed by Carsidaridae as the second branch. In contrast, concatenation methods unanimously rejected Aphalaridae monophyly, instead recovering *Aphalara* as the second diverging lineage and Carsidaridae as the third (Appendix A). Liviidae exhibited a close affinity to the (Psyllidae + Triozidae) clade across all analyses (Figure 1a; UFBoot = 100, SH‐aLRT = 100, wASTRAL = 1). However, the monophyly of Psyllidae was compromised by the unstable position of the rogue genus *Diaphorina*. In coalescent analyses and concatenation analyses based on Matrix1, *Diaphorina* was recovered as sister to the remaining Psyllidae and Triozidae, albeit with moderate support (Figure 1a; UFBoot = 99–100, SH‐aLRT = 98.9–99.9, wASTRAL = 0.45). Conversely, all concatenation analyses based on Matrix2 supported the monophyly of Psyllidae, with *Diaphorina* sister to the remaining family (Figure 1a; UFBoot = 97–99, SH‐aLRT = 91.5–99.5).

Within Aphidoidea, all analyses unanimously supported Aphididae as the sister group to the (Adelgidae + Phylloxeridae) clade with maximal support (Figure 1a; UFBoot = 100, SH‐aLRT = 100, wASTRAL = 1). Similarly, the Coccoidea phylogeny demonstrated remarkable consistency across all analytical approaches, revealing a strongly supported sister relationship between Dactylopiidae and Eriococcidae (Figure 1a; UFBoot = 100, SH‐aLRT = 100, wASTRAL = 1).

We used FcLM analyses to detect potential confounding signals with both matrices and evaluated which hypothesis was predominantly supported by these quartets: T1, (Aleyrodomorpha + Psyllomorpha) and (Coccomorpha + Aphidomorpha); T2, (Aleyrodomorpha + Aphidomorpha) and (Psyllomorpha + Coccomorpha); and T3, (Aleyrodomorpha + Coccomorpha) and (Psyllomorpha + Aphidomorpha). The majority of quartets placing Aleyrodomorpha plus Psyllomorpha were sister groups to a clade comprising (Coccomorpha + Aphidomorpha), with maximal support (Figure 1b).

## 4 Discussion

### 4.1 Systematic Position of Aleyrodoidea

The rooting of Sternorrhyncha is pivotal for understanding the evolutionary origins of this group, yet it has remained contentious. While phylogenomic studies have made significant advances, with most analyses consistently recovering Aleyrodoidea as the first diverging lineage followed by Psylloidea as the third diverging clade (Misof et al. 2014; Johnson et al. 2018; Kieran et al. 2019; Wang et al. 2019; Song and Zhang 2022), recent UCE‐based analyses have challenged this consensus by proposing an alternative topology where Aleyrodoidea forms a sister group with Psylloidea (Liu et al. 2024). This relationship was primarily supported by morphological evidence (von Dohlen and Moran 1995; Drohojowska et al. 2020).

UCEs have emerged as a powerful tool in insect phylogenetics, offering a cost‐effective and scalable alternative to whole‐genome approaches (Liu et al. 2024). The standard UCE methodology targets both exonic and intronic regions, but can only employ NT data for phylogenetic reconstruction (Faircloth et al. 2012; Liu et al. 2024). However, NT data may introduce systematic errors due to rate or compositional heterogeneity, and site saturation (Wang et al. 2023). Furthermore, the UCE matrices constructed by Liu et al. (2024) contain substantial missing data, potentially exacerbating phylogenetic uncertainty.

To address these limitations, we constructed the most comprehensive Sternorrhyncha dataset to date by integrating available transcriptomes and genomes, from which we extracted USCOs. Our analytical framework incorporated both better‐fitting site‐heterogeneous models and the MSC model to account for potential systematic biases. All analyses unanimously rejected the (Aleyrodoidea + Psylloidea) sister relationship, instead strongly supporting Aleyrodoidea as the first diverging lineage. These results corroborate previous phylogenomic studies (Misof et al. 2014; Johnson et al. 2018; Kieran et al. 2019; Wang et al. 2019; Song and Zhang 2022), suggesting that the alternative topology recovered in UCE‐based analyses may represent an artifact. Meanwhile, the FcLM analyses also supported the Aleyrodomorpha and Psyllomorpha as sister to a clade comprising Coccomorpha and Aphidomorpha (Figure 1b).

### 4.2 Phylogeny of Psylloidea

Wang et al. (2023) employed a genome‐scale approach to reconstruct the phylogeny of Psylloidea, explicitly accounting for systematic bias. By analyzing hundreds of USCOs derived from psyllid transcriptomes and genomes, their AA‐based analysis recovered the relationship (Aphalaridae, (Carsidaridae, others)) with maximal support, a topology further corroborated by their systematic‐bias‐aware NT‐based analyses.

Our coalescent analysis recovered the same topology as the ML tree inferred from concatenated AA sequences in Wang et al. (2023), though the phylogenetic placement of *Diaphorina* differed. In our MSC tree, this genus formed a sister group to the (Triozidae + Psyllidae) clade, albeit with weak support (wASTRAL = 0.45). This relationship was strongly supported by concatenation analyses based on Matrix1. However, recent classifications integrating morphological and molecular evidence (Percy et al. 2018; Burckhardt et al. 2021) place *Diaphorina* at the most ‘basal’ position within Psyllidae. In our study, the monophyly of Psyllidae was strongly supported by concatenation analyses using Matrix2, with *Diaphorina* emerging as the sister group to the remaining psyllids. Given these conflicting results, the phylogenetic position of *Diaphorina* remains uncertain and warrants further investigation through expanded taxon sampling.

Additionally, the monophyly of Aphalaridae received only moderate support, with robust confirmation solely in the coalescent analysis. In concatenation analyses, Aphalaridae was recovered as paraphyletic due to the unstable placement of *Aphalara*. While earlier studies based on Sanger sequencing or mitogenome data also suggested paraphyly for this family (Percy et al. 2018; Cho et al. 2019), genome‐scale phylogenies, particularly those accounting for systematic errors (Wang et al. 2023, with species sampling consistent with our study), strongly support its monophyly. Thus, we argue that the observed paraphyly in concatenation analyses may represent an artifact.

Regarding the phylogenetic position of Aphalaridae, Liu et al. (2024) recovered an (Aphalaridae + Carsidaridae) clade using NT data from UCEs, aligning with the NT‐based ML analysis by Wang et al. (2023). However, Wang et al. (2023) demonstrated that this relationship likely reflects systematic error stemming from compositional heterogeneity, a bias potentially mitigated by AA data or RY‐coding strategies. We concur with their conclusion that AA‐based concatenation analyses are more reliable than NT‐based approaches for resolving deep‐level relationships within Psylloidea. Our results strongly support Aphalaridae as the sister group to Carsidaridae and the remaining psylloid families, a topology consistently recovered in both concatenation and coalescent analyses.

Notably, several families (e.g., Mastigimatidae and Calophyidae) were absent from our dataset, and taxon sampling across included families remained limited, as has also been the case in previous studies (Wang et al. 2023; Liu et al. 2024; Deng et al. 2025). The monophyly of Psylloidea has been well established (Liu et al. 2024; Deng et al. 2025), and our primary focus was on the relationships between Psylloidea and other families; therefore, the limited internal sampling within Psylloidea has minimal impact on our main conclusions. Nevertheless, the backbone phylogeny of Psylloidea requires further phylogenomic interrogation, and its classification framework demands additional refinement through future research.

### 4.3 Phylogeny of Coccoidea

Coccoidea are traditionally divided into two informal groups: archaeococcoids and neococcoids. Archaeococcoids are typically characterized by the presence of abdominal spiracles in at least the females, by adult males possessing either compound or multifaceted eyes, or a row of unicorneal eyes encircling the head and by an XX‐XO sex determination system (Borchsenius 1958; Williams et al. 2011; Deng et al. 2025). Furthermore, most archaeococcoid lineages appear earlier in the fossil record than nearly all identifiable neococcoid lineages (Gullan and Cook 2007). These morphological and paleontological features collectively suggest that archaeococcoids represent a more ancestral group than neococcoids.

The scarcity of genomic data has long impeded robust phylogenetic reconstruction within Coccoidea. Recent advances in phylogenomics have addressed this gap through analyses of UCEs (Liu et al. 2024) and USCOs (Song et al. 2024; Deng et al. 2025). Notably, Deng et al. (2025) contributed the most extensive genomic dataset to date. While our analyses were completed prior to the publication of their study, and given our primary focus on Sternorrhyncha's backbone phylogeny, we were unable to incorporate their newly sequenced genomes. Our taxon sampling scheme closely resembles those of Song et al. (2024) and Liu et al. (2024).

Our phylogenetic analyses yielded highly consistent topologies for Coccoidea across multiple analytical methods and evolutionary models. In contrast to Liu et al.'s (2024) UCE‐based study that placed Dactylopiidae (*Dactylopius coccus*) within archaeococcoids, our results positioned this family within neococcoids as sister to Eriococcidae. This finding aligns with the conventional hypothesis proposing close relationships among Dactylopiidae, Beesoniidae, Stictococcidae, and certain Eriococcidae lineages (Cook et al. 2002; Cook and Gullan 2004; Gullan and Cook 2007; Hodgson and Hardy 2013; Vea and Grimaldi 2016) and has received support from recent phylogenomic studies (Song et al. 2024; Deng et al. 2025). The aberrant placement of Dactylopiidae in archaeococcoids by Liu et al. (2024) likely represents a systematic artifact arising from compositional heterogeneity in UCE data.

Additionally, our AA‐based phylogenomic analyses revealed a different systematic position for Conchaspididae compared to Liu et al. (2024). Rather than forming a sister relationship with Pseudococcidae, Conchaspididae clustered with a clade comprising all neococcoid families except Pseudococcidae. This placement is more congruent with morphological evidence: Conchaspididae and Diaspididae share several key morphological traits (e.g., tubular pores, sclerotised anal plates) not found in Pseudococcidae, and adult male characteristics of Conchaspididae align more closely with the “diaspidoid” type than with anything seen in Pseudococcidae (Mamet 1954). This instability in the placement of Conchaspididae was also observed by Song et al. (2024), where ML partitioned analyses initially recovered it as sister to remaining neococcoids, but subsequent correction for site‐specific compositional heterogeneity yielded results congruent with our study. Considering the additional support from Deng et al. (2025), we conclude that the proposed sister relationship between Conchaspididae and Pseudococcidae likely represents a methodological artifact. Despite these advances, the precise phylogenetic placement of Conchaspididae remains uncertain, and a comprehensive resolution of the phylogeny of Coccoidea will require broader taxon sampling across this enigmatic family and the highly diverse superfamily as a whole.

### 4.4 Phylogeny of Aphidoidea

Aphidoidea comprises three recognized families: Aphididae, Adelgidae, and Phylloxeridae. These groups exhibit distinct reproductive strategies, with Aphididae displaying viviparous parthenogenesis, while Adelgidae and Phylloxeridae are characterized by oviparous parthenogenesis (Liu et al. 2024). A key morphological distinction lies in the absence of siphunculi in Adelgidae and Phylloxeridae, contrasting with their presence in Aphididae (Havill et al. 2007). Phylogenetic relationships among these three families have shown remarkable consistency across studies employing various molecular datasets, including both NT and AA sequences. However, the resolution of internal relationships within the speciose family Aphididae remains problematic.

Both our concatenation analyses and previous UCE‐based studies (Liu et al. 2024) reveal exceptionally short internode branch lengths for deep‐level relationships among aphidid lineages. These compressed phylogenetic distances suggest an ancient rapid radiation event, providing a plausible explanation for the persistent challenges in reconstructing Aphididae phylogeny.

To advance our understanding of Aphididae evolution, future studies should prioritize comprehensive sampling across all extant subfamilies. Additionally, incorporating complementary lines of evidence, particularly genomic data from aphid‐specific endosymbiotic bacteria (Jousselin et al. 2024), may provide valuable phylogenetic signals. This integrative approach promises to elucidate the complex evolutionary history of this ecologically and economically significant insect group.

## Author Contributions

**Shiyu Du:** formal analysis (equal), visualization (lead), writing – original draft (equal), writing – review and editing (equal). **Yehao Wang:** methodology (lead), writing – original draft (equal), writing – review and editing (equal). **Yuang Li:** writing – review and editing (equal). **Chenyang Cai:** conceptualization (lead), funding acquisition (lead), resources (lead), writing – review and editing (equal).

## Funding

This work was supported by the National Natural Science Foundation of China (Grant 42222201).

## Conflicts of Interest

The authors declare no conflicts of interest.

## Supporting information


**Table S1:** Taxa sampling and basic statistics for USCO extraction in this study.

**Appendix A.** Summary of the phylogenetic trees constructed in this study.

## Data Availability

All data are included within the article and its Supporting Information.

## References

[ece372636-bib-0001] Borchsenius, N. S. 1958. “On the Evolution and Phylogenetic Interrelations of Coccoidea (Insecta, Homoptera).” Zoologicheskiĭ Zhurnal 37: 765–780.

[ece372636-bib-0002] Bourgoin, T. , and B. C. Campbell . 2002. Inferring a Phylogeny for Hemiptera: Falling Into the “Autapomorphic Trap”, 67–82. Denisia 4.

[ece372636-bib-0003] Burckhardt, D. , D. Ouvrard , and D. M. Percy . 2021. “An Updated Classification of the Jumping Plant‐Lice (Hemiptera: Psylloidea) Integrating Molecular and Morphological Evidence.” European Journal of Taxonomy 736: 137–182.

[ece372636-bib-0004] Campbell, B. C. , J. D. Steffen‐Campbell , and R. J. Gill . 1994. “Evolutionary Origin of Whiteflies (Hemiptera: Sternorrhyncha: Aleyrodidae) Inferred From 18s rDNA Sequences.” Insect Molecular Biology 3, no. 2: 73–88. 7987524 10.1111/j.1365-2583.1994.tb00154.x

[ece372636-bib-0005] Chernomor, Q. , A. von Haeseler , and B. Q. Minh . 2016. “Terrace Aware Data Structure for Phylogenomic Inference From Supermatrices.” Systematic Biology 65: 997–1008. 27121966 10.1093/sysbio/syw037 PMC5066062

[ece372636-bib-0006] Cho, G. , I. Malenovský , and S. Lee . 2019. “Higher‐Level Molecular Phylogeny of Jumping Plant Lice (Hemiptera: Sternorrhyncha: Psylloidea).” Systematic Entomology 44, no. 3: 638–651.

[ece372636-bib-0007] Cook, L. G. , and P. J. Gullan . 2004. “The Gall‐Inducing Habit Has Evolved Multiple Times Among the Eriococcid Scale Insects (Sternorrhyncha: Coccoidea: Eriococcidae).” Biological Journal of the Linnean Society 83: 441–452.

[ece372636-bib-0008] Cook, L. G. , P. J. Gullan , and H. E. Trueman . 2002. “A Preliminary Phylogeny of the Scale Insects (Hemiptera: Sternorrhyncha: Coccoidea) Based on Nuclear Small‐Subunit Ribosomal DNA.” Molecular Phylogenetics and Evolution 25: 43–52. 12383749 10.1016/s1055-7903(02)00248-8

[ece372636-bib-0009] Criscuolo, A. , and S. Gribaldo . 2010. “BMGE (Block Mapping and Gathering With Entropy): A New Software for Selection of Phylogenetic Informative Regions From Multiple Sequence Alignments.” BMC Evolutionary Biology 10: 210. 20626897 10.1186/1471-2148-10-210 PMC3017758

[ece372636-bib-0010] Cryan, J. R. , and J. M. Urban . 2012. “Higher‐Level Phylogeny of the Insect Order Hemiptera: Is Auchenorrhyncha Really Paraphyletic?” Systematic Entomology 37: 7–21.

[ece372636-bib-0011] Deng, J. , X. Weng , W. Ma , et al. 2025. “Genomic Insights Into the Phylogeny and Evolutionary History of Scale Insects (Hemiptera: Coccoidea): Resolving Family‐Level Relationships.” Molecular Phylogenetics and Evolution 210: 108383. 40451495 10.1016/j.ympev.2025.108383

[ece372636-bib-0012] Drohojowska, J. , J. Szwedo , D. Żyła , D. Y. Huang , and P. Müller . 2020. “Fossils Reshape the Sternorrhyncha Evolutionary Tree (Insecta, Hemiptera).” Scientific Reports 10, no. 1: 11390. 32647332 10.1038/s41598-020-68220-x PMC7347605

[ece372636-bib-0013] Du, S. , E. Tihelka , D. Yu , et al. 2024. “Revisiting the Four Hexapoda Classes: Protura as the Sister Group to All Other Hexapods.” Proceedings of the National Academy of Sciences of the United States of America 121, no. 39: e2408775121. 39298489 10.1073/pnas.2408775121 PMC11441524

[ece372636-bib-0014] Faircloth, B. C. , J. E. McCormack , N. G. Crawford , M. G. Harvey , R. T. Brumfield , and T. C. Glenn . 2012. “Ultraconserved Elements Anchor Thousands of Genetic Markers Spanning Multiple Evolutionary Timescales.” Systematic Biology 61, no. 5: 717–726. 22232343 10.1093/sysbio/sys004

[ece372636-bib-0015] Feuda, R. , M. Dohrmann , W. Pett , et al. 2017. “Improved Modeling of Compositional Heterogeneity Supports Sponges as Sister to All Other Animals.” Current Biology 27, no. 24: 3864–3870. 29199080 10.1016/j.cub.2017.11.008

[ece372636-bib-0016] Guindon, S. , J. F. Dufayard , V. Lefort , M. Anisimova , W. Hordijk , and O. Gascuel . 2010. “New Algorithms and Methods to Estimate Maximum‐Likelihood Phylogenies: Assessing the Performance of PhyML 3.0.” Systematic Biology 59: 307–321. 20525638 10.1093/sysbio/syq010

[ece372636-bib-0017] Gullan, P. J. , and L. G. Cook . 2007. “Phylogeny and Higher Classification of the Scale Insects (Hemiptera: Sternorrhyncha: Coccoidea).” Zootaxa 1668: 413–425.

[ece372636-bib-0018] Havill, N. P. , R. G. Foottit , and C. D. von Dohlen . 2007. “Evolution of Host Specialization in the Adelgidae (Insecta: Hemiptera) Inferred From Molecular Phylogenetics.” Molecular Phylogenetics and Evolution 44: 357–370. 17196838 10.1016/j.ympev.2006.11.008

[ece372636-bib-0019] Hoang, D. T. , O. Chernomor , A. von Haeseler , B. Q. Minh , and L. S. Vinh . 2018. “UFBoot2: Improving the Ultrafast Bootstrap Approximation.” Molecular Biology and Evolution 35, no. 2: 518–522. 29077904 10.1093/molbev/msx281 PMC5850222

[ece372636-bib-0020] Hodgson, C. J. , and N. B. Hardy . 2013. “The Phylogeny of the Superfamily Coccoidea (Hemiptera: Sternorrhyncha) Based on the Morphology of Extant and Extinct Macropterous Males.” Systematic Entomology 38: 794–804.

[ece372636-bib-0021] Johnson, K. P. , C. H. Dietrich , F. Friedrich , et al. 2018. “Phylogenomics and the Evolution of Hemipteroid Insects.” Proceedings of the National Academy of Sciences 115, no. 50: 12775–12780. 10.1073/pnas.1815820115 PMC6294958 30478043

[ece372636-bib-0022] Jousselin, E. , A. Coeur d'acier , A. L. Clamens , et al. 2024. “Discordance Between Mitochondrial, Nuclear, and Symbiont Genomes in Aphid Phylogenetics: Who Is Telling the Truth?” Zoological Journal of the Linnean Society 201, no. 4: zlae098.

[ece372636-bib-0023] Kalyaanamoorthy, S. , B. Q. Minh , T. K. F. Wong , A. von Haeseler , and L. S. Jermiin . 2017. “ModelFinder: Fast Model Selection for Accurate Phylogenetic Estimates.” Nature Methods 14: 587–589. 28481363 10.1038/nmeth.4285 PMC5453245

[ece372636-bib-0024] Kieran, T. J. , E. R. L. Gordon , M. Forthman , et al. 2019. “Insight From an Ultraconserved Element Bait Set Designed for Hemipteran Phylogenetics Integrated With Genomic Resources.” Molecular Phylogenetics and Evolution 130: 297–303. 30359745 10.1016/j.ympev.2018.10.026

[ece372636-bib-0025] Kriventseva, E. V. , D. Kuznetsov , F. Tegenfeldt , et al. 2019. “OrthoDB v10: Sampling the Diversity of Animal, Plant, Fungal, Protist, Bacterial and Viral Genomes for Evolutionary and Functional Annotations of Orthologs.” Nucleic Acids Research 47, no. D1: D807–D811. 30395283 10.1093/nar/gky1053 PMC6323947

[ece372636-bib-0026] Kück, P. , and G. C. Longo . 2014. “FASconCAT‐G: Extensive Functions for Multiple Sequence Alignment Preparations Concerning Phylogenetic Studies.” Frontiers in Zoology 11: 81. 25426157 10.1186/s12983-014-0081-x PMC4243772

[ece372636-bib-0027] Le, S. Q. , C. C. Dang , and O. Gascuel . 2012. “Modeling Protein Evolution With Several Amino Acid Replacement Matrices Depending on Site Rates.” Molecular Biology and Evolution 29, no. 10: 2921–2936. 22491036 10.1093/molbev/mss112

[ece372636-bib-0028] Letunic, I. , and P. Bork . 2024. “Interactive Tree of Life (iTOL) v6: Recent Updates to the Phylogenetic Tree Display and Annotation Tool.” Nucleic Acids Research 52: W78–W82. 38613393 10.1093/nar/gkae268 PMC11223838

[ece372636-bib-0029] Li, H. , J. M. Leavengood , E. G. Chapman , et al. 2017. “Mitochondrial Phylogenomics of Hemiptera Reveals Adaptive Innovations Driving the Diversification of True Bugs.” Proceedings of the Royal Society B: Biological Sciences 284, no. 1862: 20171223. 10.1098/rspb.2017.1223 PMC5597834 28878063

[ece372636-bib-0030] Li, W. , L. Jaroszewski , and A. Godzik . 2002. “Tolerating Some Redundancy Significantly Speeds Up Clustering of Large Protein Databases.” Bioinformatics 18, no. 1: 77–82. 11836214 10.1093/bioinformatics/18.1.77

[ece372636-bib-0031] Liu, D. , J. Cui , Y. Liu , et al. 2024. “Ultraconserved Elements From Transcriptome and Genome Data Provide Insight Into the Phylogenomics of Sternorrhyncha (Insecta: Hemiptera).” Cladistics 40, no. 5: 496–509. 38808591 10.1111/cla.12585

[ece372636-bib-0032] Mamet, J. R. 1954. “A Monograph of the Conchaspididae Green (Hemiptera: Coccoidea).” Transactions of the Royal Entomological Society of London 105: 189–239.

[ece372636-bib-0033] Manni, M. , M. R. Berkeley , M. Seppey , F. A. Simão , and E. M. Zdobnov . 2021. “BUSCO Update: Novel and Streamlined Workflows Along With Broader and Deeper Phylogenetic Coverage for Scoring of Eukaryotic, Prokaryotic, and Viral Genomes.” Molecular Biology and Evolution 38, no. 10: 4647–4654. 34320186 10.1093/molbev/msab199 PMC8476166

[ece372636-bib-0034] Minh, B. Q. , H. A. Schmidt , Q. Chernomor , et al. 2020. “IQ‐TREE 2: New Models and Efficient Methods for Phylogenetic Inference in the Genomic Era.” Molecular Biology and Evolution 37, no. 5: 1530–1534. 32011700 10.1093/molbev/msaa015 PMC7182206

[ece372636-bib-0035] Misof, B. , S. Liu , K. Meusemann , et al. 2014. “Phylogenomics Resolves the Timing and Pattern of Insect Evolution.” Science 346, no. 6210: 763–767. 25378627 10.1126/science.1257570

[ece372636-bib-0036] Percy, D. M. , A. Crampton‐Platt , S. Sveinsson , et al. 2018. “Resolving the Psyllid Tree of Life: Phylogenomic Analyses of the Superfamily Psylloidea (Hemiptera).” Systematic Entomology 43, no. 4: 762–776.

[ece372636-bib-0037] Philippe, H. , A. J. Poustka , M. Chiodin , et al. 2019. “Mitigating Anticipated Effects of Systematic Errors Supports Sister‐Group Relationship Between Xenacoelomorpha and Ambulacraria.” 29, no. 11: 1818–1826. 10.1016/j.cub.2019.04.009 31104936

[ece372636-bib-0038] Prjibelski, A. , D. Antipov , D. Meleshko , A. Lapidus , and A. Korobeynikov . 2020. “Using SPAdes De Novo Assembler.” Current Protocols in Bioinformatics 70: e102. 32559359 10.1002/cpbi.102

[ece372636-bib-0039] Ranwez, V. , E. J. P. Douzery , C. Cambon , N. Chantret , and F. Delsuc . 2018. “MACSE v2: Toolkit for the Alignment of Coding Sequences Accounting for Frameshifts and Stop Codons.” Molecular Biology and Evolution 35, no. 10: 2582–2584. 30165589 10.1093/molbev/msy159 PMC6188553

[ece372636-bib-0040] Simion, P. , H. Philippe , D. Baurain , et al. 2017. “A Large and Consistent Phylogenomic Dataset Supports Sponges as the Sister Group to All Other Animals.” Current Biology 27, no. 7: 958–967. 28318975 10.1016/j.cub.2017.02.031

[ece372636-bib-0041] Song, N. , M. Wang , H. Tang , and Z. Dang . 2024. “A Phylogenetic Analysis of Scale Insects (Hemiptera, Coccoidea) Based on Genomic and Transcriptomic Data.” Journal of Systematics and Evolution 63, no. 3: 693–707.

[ece372636-bib-0042] Song, N. , and H. Zhang . 2022. “A Comprehensive Analysis of Higher‐Level Phylogenetic Relationships of Hemiptera Based on Transcriptome Data.” Journal of Systematics and Evolution 61, no. 4: 572–586.

[ece372636-bib-0043] Strimmer, K. , and A. von Haeseler . 1997. “Likelihood‐Mapping: A Simple Method to Visualize Phylogenetic Content of a Sequence Alignment.” Proceedings of the National Academy of Sciences 94: 6815–6819. 10.1073/pnas.94.13.6815 PMC21241 9192648

[ece372636-bib-0044] Vea, I. M. , and D. A. Grimaldi . 2016. “Putting Scales Into Evolutionary Time: The Divergence of Major Scale Insect Lineages (Hemiptera) Predates the Radiation of Modern Angiosperm Hosts.” Scientific Reports 6: 23487. 27000526 10.1038/srep23487 PMC4802209

[ece372636-bib-0045] von Dohlen, C. D. , and N. A. Moran . 1995. “Molecular Phylogeny of the Homoptera: A Paraphyletic Taxon.” Journal of Molecular Evolution 41: 211–223. 7666451 10.1007/BF00170675

[ece372636-bib-0046] Wang, H. C. , B. Q. Minh , S. Susko , and A. J. Roger . 2018. “Modeling Site Heterogeneity With Posterior Mean Site Frequency Profiles Accelerates Accurate Phylogenomic Estimation.” Systematic Biology 67: 216–235. 28950365 10.1093/sysbio/syx068

[ece372636-bib-0047] Wang, W. , Z. Dong , Z. Du , and P. Wu . 2023. “Genome‐Scale Approach to Reconstructing the Phylogenetic Tree of Psyllids (Superfamily Psylloidea) With Account of Systematic Bias.” Molecular Phylogenetics and Evolution 189: 107924. 37699449 10.1016/j.ympev.2023.107924

[ece372636-bib-0048] Wang, Y. , H. Wu , D. Rédei , et al. 2019. “When Did the Ancestor of True Bugs Become Stinky? Disentangling the Phylogenomics of Hemiptera–Heteroptera.” Cladistics 35: 42–66. 34636080 10.1111/cla.12232

[ece372636-bib-0049] Waterhouse, R. M. , M. Seppey , F. A. Simão , et al. 2017. “BUSCO Applications From Quality Assessments to Gene Prediction and Phylogenomics.” Molecular Biology and Evolution 35, no. 3: 543–548. 10.1093/molbev/msx319 PMC5850278 29220515

[ece372636-bib-0050] Williams, D. J. , P. J. Gullan , D. R. Miller , D. Matile‐Ferrero , and S. I. Han . 2011. “A Study of the Scale Insect Genera *Puto* Signoret (Hemiptera: Sternorrhyncha: Coccoidea: Putoidae) and *Ceroputo* Sulc (Pseudococcidae) With a Comparison to *Phenacoccus* Cockerell (Pseudococcidae).” Zootaxa 2802: 1–22.

[ece372636-bib-0051] Williams, T. A. , C. J. Cox , P. G. Foster , G. J. Szöllősi , and T. M. Embley . 2020. “Phylogenomics Provides Robust Support for a Two‐Domains Tree of Life.” Nature Ecology & Evolution 4: 138–147. 31819234 10.1038/s41559-019-1040-x PMC6942926

[ece372636-bib-0052] Xiong, X. , L. Wu , T. Xin , J. Wang , Z. Zou , and B. Xia . 2017. “The Complete Mitochondrial Genome of *Diaphorina citri* (Hemiptera: Psyllidae) and Phylogenetic Analysis.” Biochemical Systematics and Ecology 70: 230–238.

[ece372636-bib-0053] Zhang, C. , R. Nielsen , and S. Mirarab . 2025. “ASTER: A Package for Large‐Scale Phylogenomic Reconstructions.” Molecular Biology and Evolution 42, no. 8: msaf172. 40668947 10.1093/molbev/msaf172 PMC12343031

[ece372636-bib-0054] Zhou, Q. , M. Xiong , A. Luo , X. Wang , and S. Wu . 2022. “Characterization of the First Mitochondrial Genome of Aclerdidae (Hemiptera: Coccoidea) With a Novel Gene Arrangement.” Zoological Systematics 47, no. 4: 293–304.

