# Supplementary figures and images for: Phylogenomics Resolves the Evolution of Sternorrhyncha (Insecta: Hemiptera)
Source: Ecol Evol. 2025 Dec 5;15(12):e72636. doi: 10.1002/ece3.72636 (PMC12678864; doi:10.1002/ece3.72636)

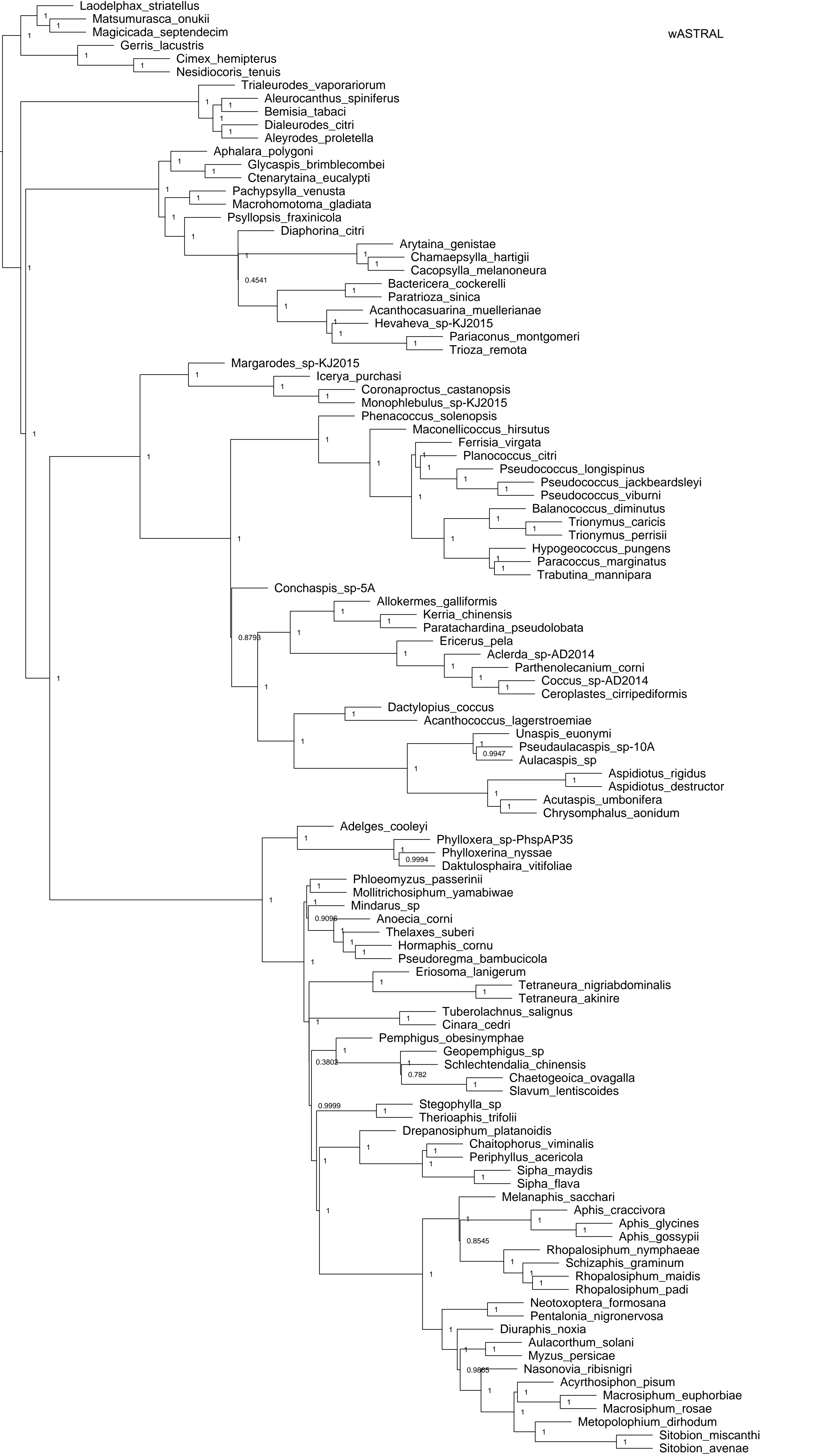

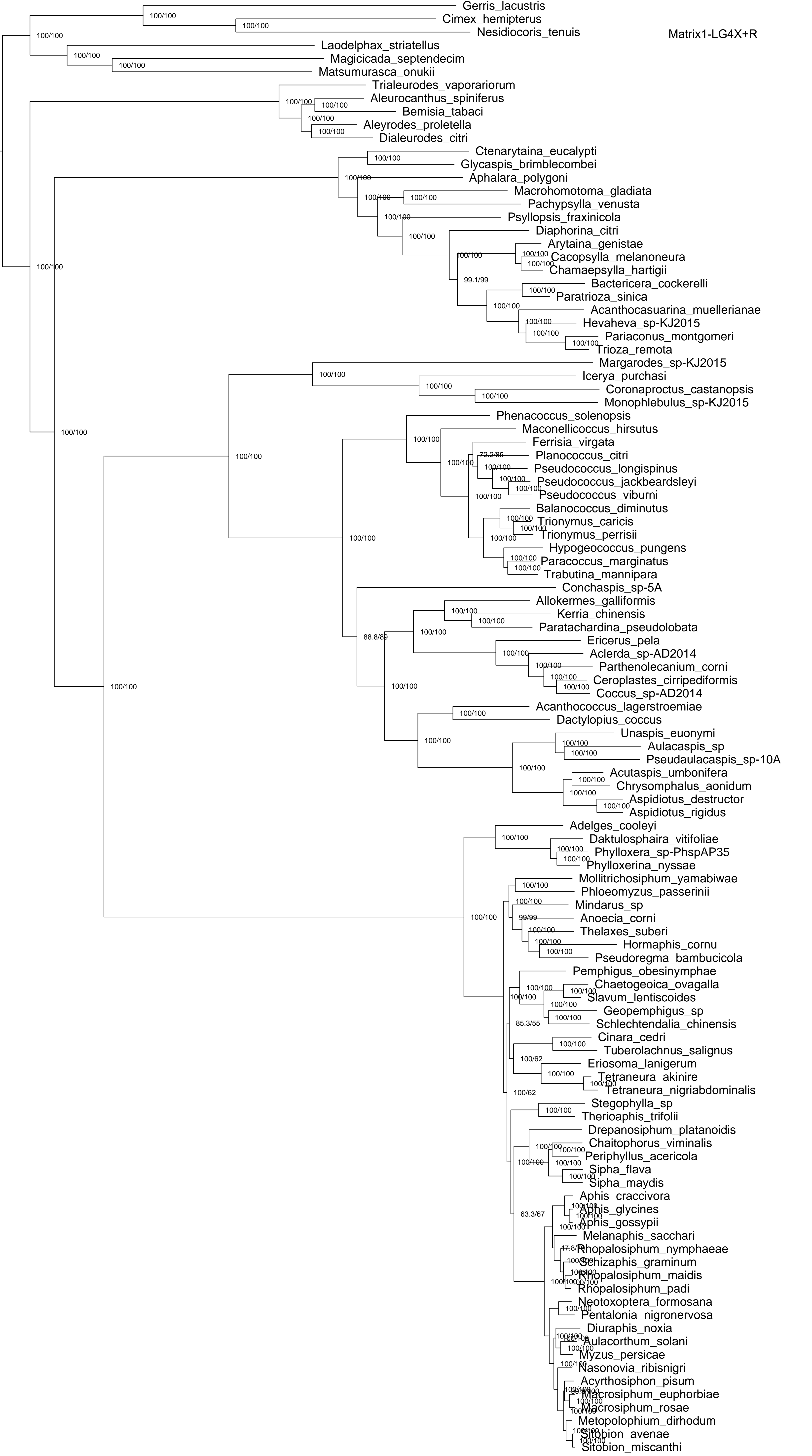

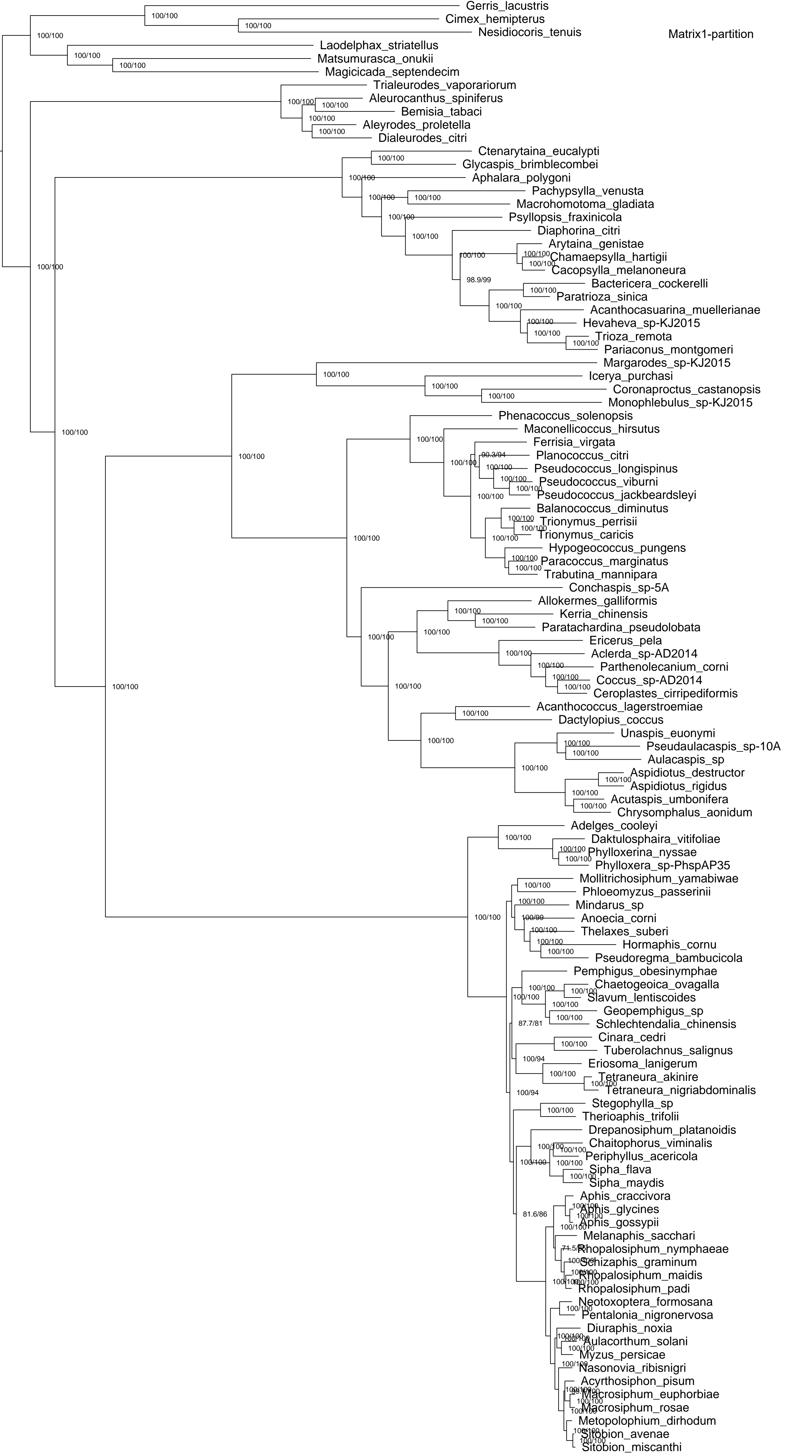

Matrix1-partition

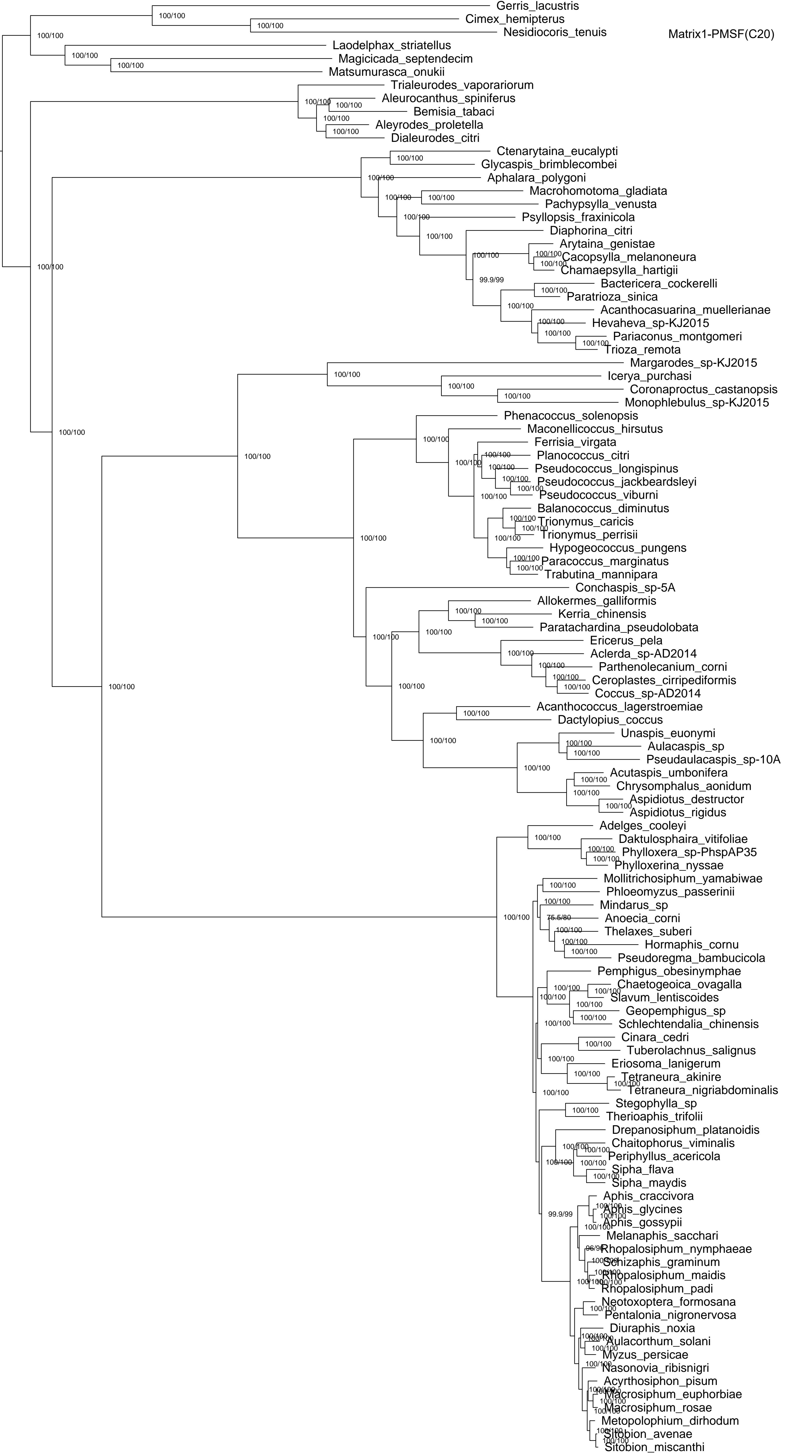

Matrix1-PMSF(C20)

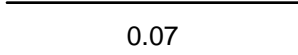

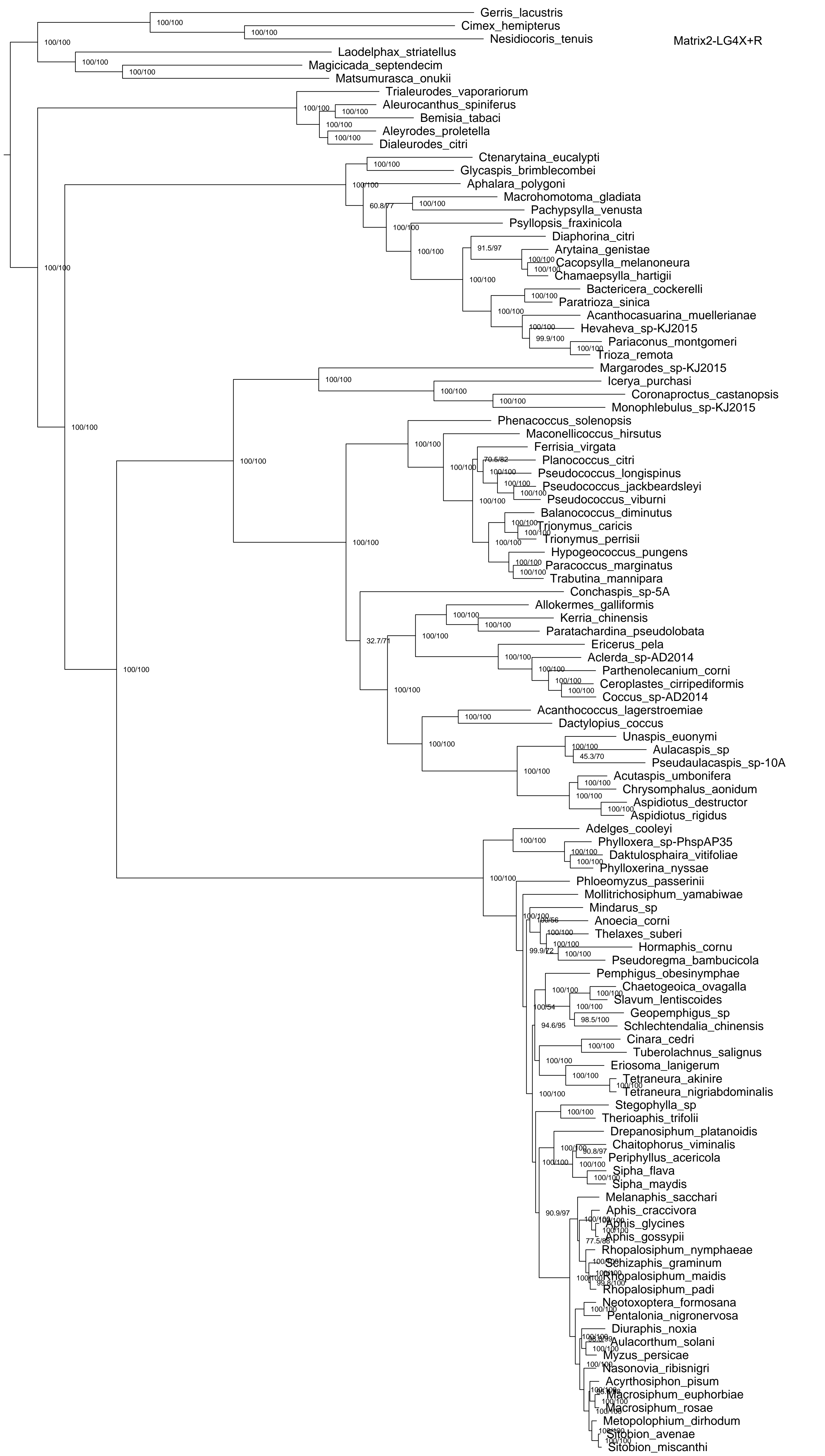

Matrix2-LG4X+R

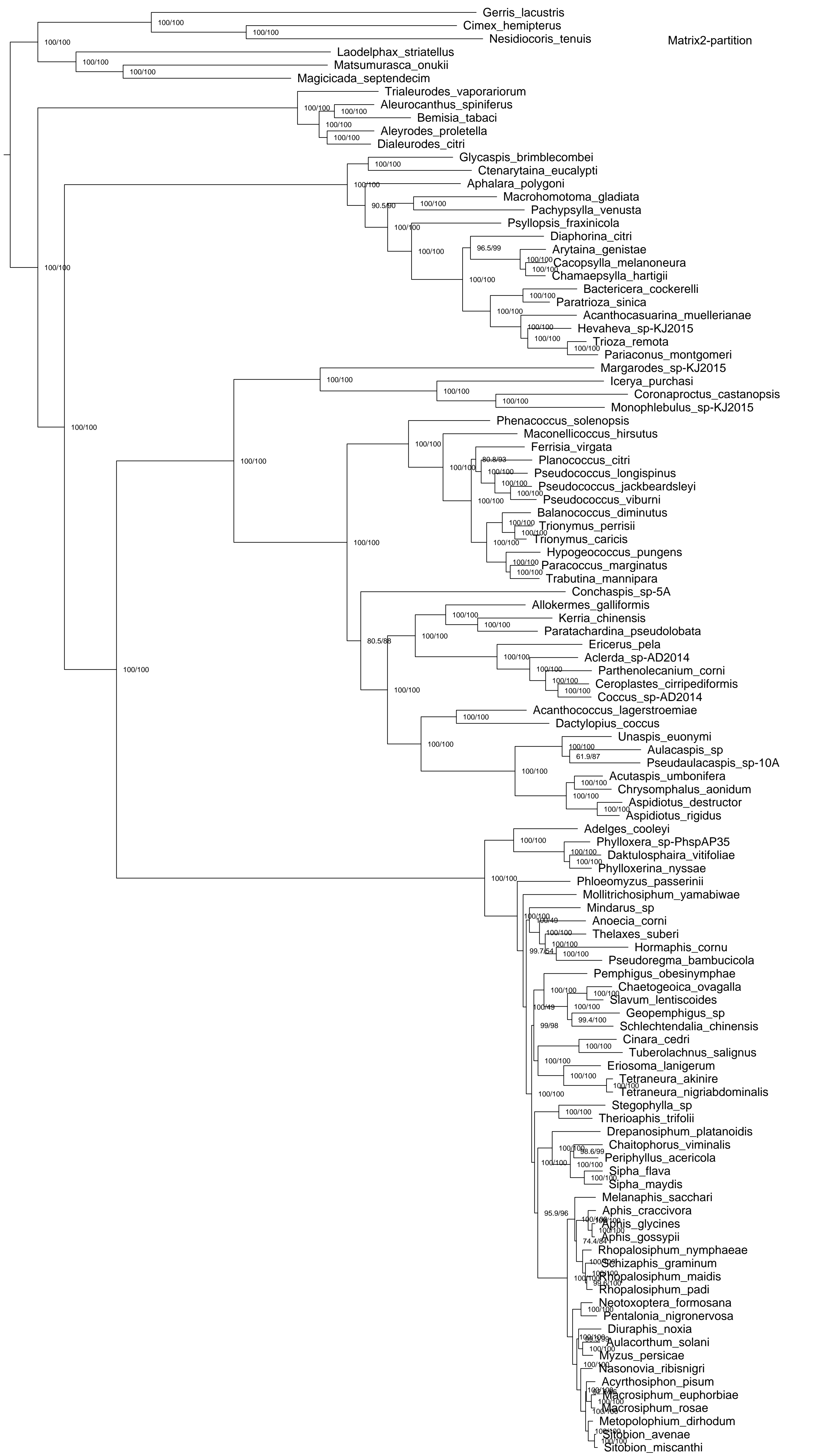

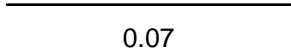

Supplement: Supplementary file 2 — Appendix A. Summary of the phylogenetic trees constructed in this study. [file ECE3-15-e72636-s001.pdf]
